# Protein-truncating variant in APOL3 increases chronic kidney disease risk in epistasis with APOL1 risk alleles

**DOI:** 10.1172/jci.insight.181238

**Published:** 2024-10-08

**Authors:** David Y. Zhang, Michael G. Levin, Jeffrey T. Duda, Latrice G. Landry, Walter R. Witschey, Scott M. Damrauer, Marylyn D. Ritchie, Daniel J. Rader

**Affiliations:** 1 Department of Genetics,; 2 Department of Medicine, and; 3 Division of Cardiovascular Medicine, Perelman School of Medicine, University of Pennsylvania, Philadelphia, Pennsylvania, USA.; 4 Corporal Michael J. Crescenz VA Medical Center, Philadelphia, Pennsylvania, USA.; 5 Penn Image Computing and Science Laboratory, University of Pennsylvania, Philadelphia, Pennsylvania, USA.; 6 Department of Radiology, Perelman School of Medicine, University of Pennsylvania, Philadelphia, Pennsylvania, USA.; 7 Department of Surgery, University of Pennsylvania, and; 8 Institute for Biomedical Informatics, Perelman School of Medicine, University of Pennsylvania, Philadelphia, Pennsylvania, USA.

**Keywords:** Genetics, Nephrology, Chronic kidney disease, Genetic variation

## Abstract

**BACKGROUND:**

Two coding alleles within the *APOL1* gene, G1 and G2, found almost exclusively in individuals genetically similar to West African populations, contribute substantially to the pathogenesis of chronic kidney disease (CKD). The *APOL* gene cluster on chromosome 22 contains a total of 6 *APOL* genes that have arisen as a result of gene duplication.

**METHODS:**

Using a genome-first approach in the Penn Medicine BioBank, we identified 62 protein-altering variants in the 6 *APOL* genes with a minor allele frequency of >0.1% in a population of participants genetically similar to African reference populations and performed population-specific phenome-wide association studies.

**RESULTS:**

We identified rs1108978, a stop-gain variant in *APOL3* (p.Q58*)*,* to be significantly associated with increased CKD risk, even after conditioning on *APOL1* G1/G2 carrier status. These findings were replicated in the Veterans Affairs Million Veteran Program and the *All of Us* Research Program. *APOL3* p.Q58* was also significantly associated with a number of quantitative traits linked to CKD, including decreased kidney volume. This truncating variant contributed the most risk for CKD in patients monoallelic for *APOL1* G1/G2, suggesting an epistatic interaction and a potential protective effect of wild-type APOL3 against *APOL1*-induced kidney disease.

**CONCLUSION:**

This study demonstrates the utility of targeting population-specific variants in a genome-first approach, even in the context of well-studied gene-disease relationships.

**FUNDING:**

National Heart, Lung, and Blood Institute (F30HL172382, R01HL169378, R01HL169458), Doris Duke Foundation (grant 2023-2024), National Institute of Biomedical Imaging and Bioengineering (P41EB029460), and National Center for Advancing Translational Sciences (UL1-TR-001878).

## Abstract 2

The common coding variant APOL3 p.Q58* is a genetic modifier of chronic kidney disease risk in individuals monoallelic for APOL1 G1/G2 carrier status in individuals of African ancestry.

## Introduction

Chronic kidney disease (CKD) and end-stage renal disease (ESRD) are significantly more common in individuals genetically similar to African reference populations (AFR) compared with those genetically similar to European reference populations (EUR) (1, 2). While there are multiple attributable factors, one of the most impactful is the common genetic variation in the *APOL1* gene (3–5). Two coding alleles within the *APOL1* gene, G1 and G2, are found almost exclusively in individuals genetically similar to West African populations and contribute substantially to the pathogenesis of nondiabetic kidney disease, focal segmental glomerulosclerosis, and HIV-associated nephropathy (6, 7). The G1 allele (minor allele frequency [MAF] of ~23% in individuals genetically similar to AFR used by gnomAD) comprises 2 missense variants in near-perfect linkage disequilibrium (LD), G1^G^ (p.S342G) and G1^M^ (p.I384M). The G2 allele (MAF of ~14% in individuals genetically similar to AFR used by gnomAD) is a 6-base pair in-frame deletion (p.NYK388-389K). The high allele frequency of these variants, particularly in West African populations, is caused by a recent positive selective sweep due to the protective effects they confer against *Trypanosoma brucei* infections, the cause of African sleeping sickness (8). In fact, evidence suggests that G1 and G2 are toxic gain-of-function variants in *APOL1*, a gene shown to play roles in programmed cell death and pathogen immunity (9). The G1 and G2 alleles arose independently on separate chromosomes and are too close in proximity to have undergone a recombination event that would allow a single haplotype to carry both G1 and G2. Therefore, risk of CKD is modeled on a scale of 0 to 2 by the total number of G1/G2 alleles an individual carries. Individuals with 2 G1/G2 risk alleles are considered to be “high risk” for CKD. Recently, a missense variant, p.N264K, in *APOL1* was shown to exert protective effects in high-risk individuals carrying the *APOL1* variants by inhibiting APOL1 pore-forming function and ion channel conduction (10).

The *APOL* gene cluster on chromosome 22 contains a total of 6 *APOL* genes that have arisen as a result of gene duplication (11). The physiological functions of the APOL proteins are poorly understood. We hypothesized that there may be other protein-altering variants in the *APOL* genes that are associated with health and disease in the AFR population. Adopting a genome-first approach, we leveraged the Penn Medicine BioBank (PMBB), a large medical biobank with whole-exome sequence data linked to electronic health records (EHRs) (12), to study the phenotypes associated with protein-altering variants in the 6 *APOL* genes with a focus on the AFR population. We identified an AFR-specific protein-truncating variant in *APOL3* (MAF of ~22% in individuals genetically similar to AFR used by gnomAD) that was significantly associated with increased risk of CKD and primarily increased CKD risk in monoallelic carriers of the *APOL1* G1/G2 alleles.

## Results

### Phenome-wide association studies for protein-altering variants in APOL genes.

**Figure 1:**
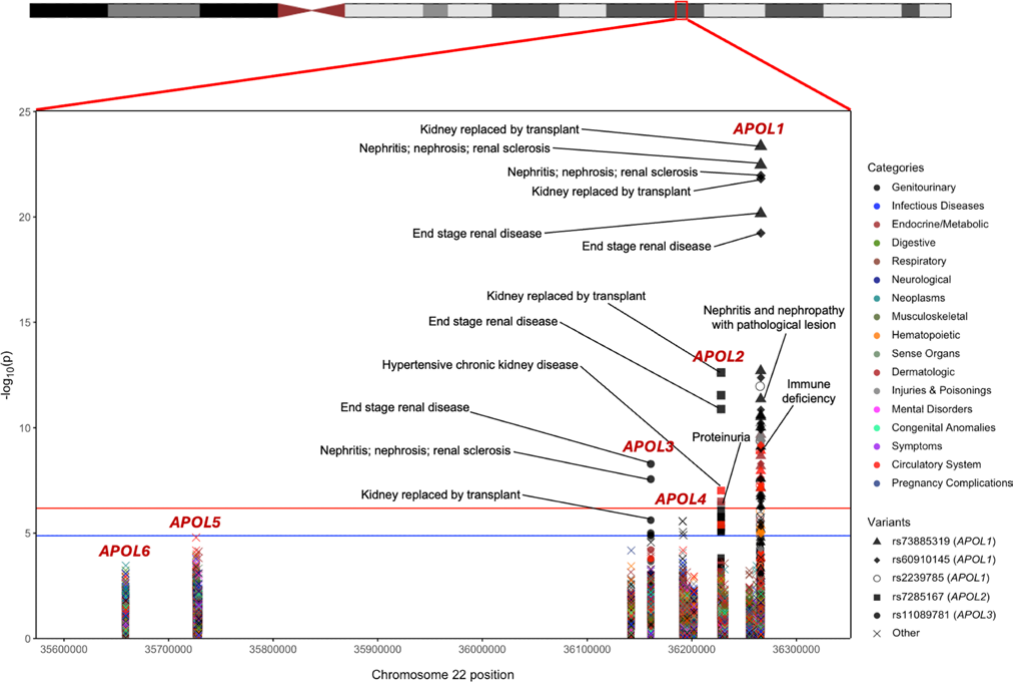
PheWAS for 62 protein-altering variants in *APOL* genes. Associations were performed in the PMBB AFR population. The red line represents the Bonferroni-adjusted *P* value significance threshold of 6.63 × 10^–7^. The blue line represents a suggestive *P* value threshold of 1.33 × 10^–5^. Variants with at least one phenotype association above the significance threshold are listed in the legend and detailed in Supplemental Table 2.

Of the 43,731 consented individuals in the PMBB with whole-exome sequencing (WES), we extracted 841 protein-altering variants across all 6 *APOL* genes, of which 100 were predicted loss of function (pLOF), 4 were in-frame insertions/deletions, and 737 were missense. With a specific focus on variants in individuals genetically similar to AFR reference populations, we filtered our variant set down to 62 variants with a MAF of 0.1% in the PMBB AFR population (*n* = 11,198), for which we show that statistical power to detect an association was sufficient (Supplemental Figure 1; supplemental material available online with this article; https://doi.org/10.1172/jci.insight.181238DS1). This set of variants included 6 pLOFs, 1 in-frame deletion, and 55 missense mutations, including both *APOL1* G1 and G2 AFR-specific risk alleles for kidney disease (Supplemental Table 1). For each of these 62 variants, we performed a phenome-wide association study (PheWAS) in the AFR population in PMBB against 1,222 binary phenotypes defined as Phecodes derived from EHR data (Figure 1), followed by additional downstream variant-specific analyses (Supplemental Figure 2).

Using a strict Bonferroni’s *P* value correction adjusting for every single variant-phenotype association performed, 58 significant associations (*P* < 6.63 × 10^–7^) were observed across 5 variants (Table 1) as were 23 unique phenotypes, all of which were related to renal disease (Supplemental Table 2). Three of the 5 significant variants were in *APOL1,* including the G1 risk allele (rs73885319 [p.S342G] and rs60910145 [p.I384M]) as well as a third missense variant rs2239785 (p.E150K). All 3 variants had strong associations with end-stage renal disease (ESRD) with odds ratios (ORs) of 1.70 (95% CI = 1.52–1.90, *P* = 6.92 × 10^–21^), 1.68 (95% CI = 1.50–1.88, *P* = 5.83 × 10^–20^), and 1.50 (95% CI = 1.34–1.68, *P* = 1.06 × 10^–12^), respectively. A missense variant in *APOL2*, rs7285167 (p.R182C), was significantly associated with ESRD with an OR of 1.43 (95% CI = 1.29–1.59, *P* = 1.29 × 10^–11^). Finally, a stop-gain variant in *APOL3*, rs11089781 (p.Q58*), was significantly associated with ESRD, with an OR of 1.39 (95% CI = 1.24–1.56, *P* = 5.18 × 10^–9^). Of note, the *APOL1* G2 allele alone did not meet our strict significance threshold but was also found to be strongly associated with ESRD with an OR of 1.33 (95% CI = 1.16-1.53, *P* = 2.45 × 10^–5^). Similarly, the *APOL1* p.N264K protective variant also did not meet our significance threshold but was found to be nominally associated with decreased risk of nephritis, nephrosis, and renal sclerosis, with an OR of 0.43 (95% CI = 0.23–0.81, *P* = 4.63 × 10^–3^), and decreased risk of ESRD, with an OR of 0.64 (95% CI = 0.41–0.99, *P* = 2.18 × 10^–2^). We repeated the analyses in the EUR population and meta-analyzed under a fixed-effects model and observed similar significant findings (Supplemental Figure 3 and Supplemental Table 3). ESRD had a prevalence of 8.4% in the PMBB AFR population. The average age of individuals with ESRD was 55.8 years old compared with 51.2 years old for individuals acting as controls (*t* test, *P* = 1.86 × 10^–22^). 58.3% of individuals in the case group were male compared with 34.8% of individuals acting as controls (χ^2^, *P* = 3.31 × 10^–45^).

**Figure 2:**
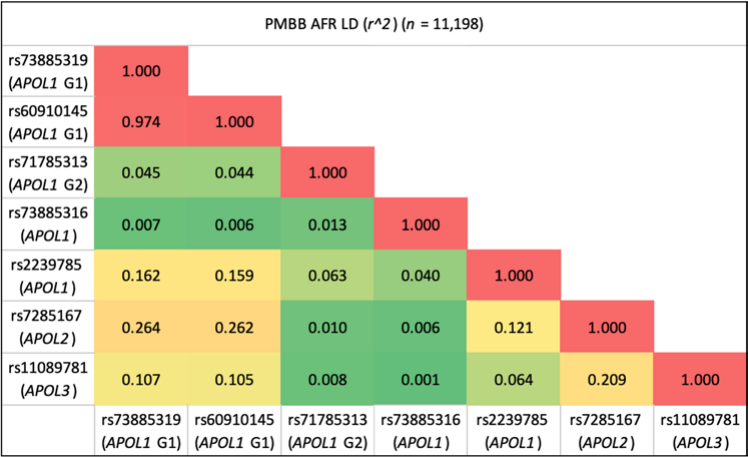
LD heatmap between significant variants in *APOL* genes. The 5 variants with significant phenotype associations as well as the *APOL1* G1/G2 risk alleles and the *APOL1* p.N264K variant. Metrics calculated using haplotypes in the PMBB AFR population (*n* = 11,198). LD metrics computed using haplotypes from the 1000 Genomes Project can be found in Supplemental Figure 4.

**Figure 3:**
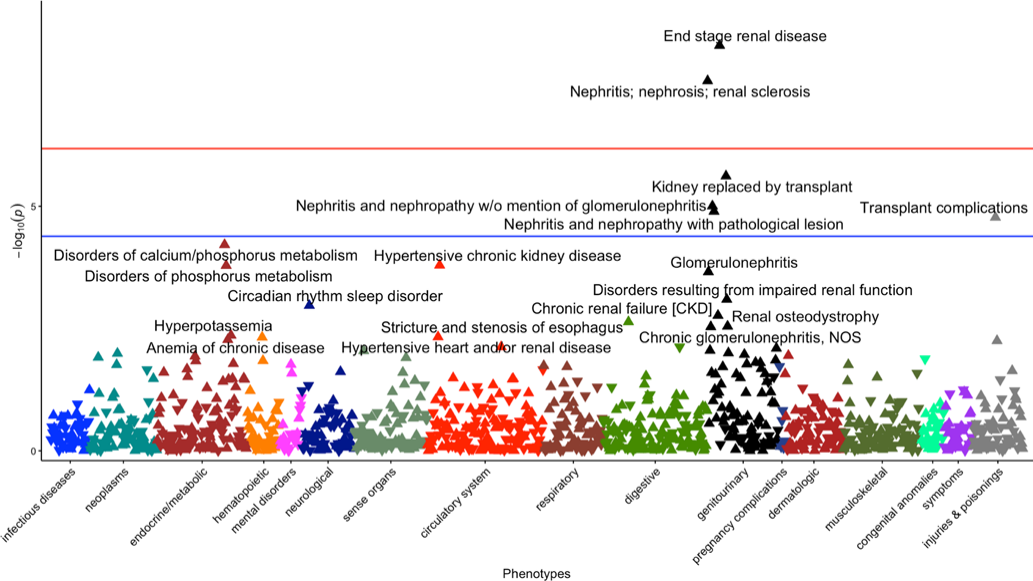
Single-variant PheWAS for *APOL3* variant rs11089781 in the PMBB AFR population. The red line represents the Bonferroni-adjusted *P* value significance threshold (*P* = 6.63 × 10^–7^) from the complete AFR associations of all 62 variants. The blue line represents the Bonferroni-adjusted *P* value significance threshold for the single-variant PheWAS of 4.11 × 10^–5^.

All 5 significant variants were substantially more common in the AFR population compared with the EUR population in PMBB, and the MAFs in PMBB were similar to the MAFs in the gnomAD v.4.0.0 database (Table 1). We examined the LD structure between these 5 variants (as well as the *APOL1* G2 allele and *APOL1* p.N264K variant) in the PMBB AFR population (Figure 2). As expected, the 2 missense variants that comprise the G1 allele were in virtually complete LD, and neither were in any LD with the G2 allele. The *APOL1* rs2239785 (p.E150K) allele had an *r^2^* value of 0.16 with the G1 allele. The *APOL2* rs7285167 (p.R182C) allele was in slightly more LD with the G1 allele (*r^2^* = 0.26). Importantly, the *APOL3* stop-gain variant, rs11089781 (p.Q58*), was in weak LD with the G1 allele (*r^2^* = 0.11) as well as with *APOL1* p.N264K (*r^2^* = 0.001). The *APOL3* variant is also highly specific for the AFR population with a PMBB AFR (*n* = 11,198) MAF of 0.211 and a PMBB EUR (*n* = 30,324) MAF of 6.43 × 10^–4^. Given that this variant appeared to have an independent signal for association with renal disease, we performed a single variant PheWAS restricted to AFR individuals, showing a strong clustering of significant kidney-associated phenotypes (Figure 3). We replicated this observed association between the *APOL3* p.Q58* variant and CKD/ESRD using identical methods in the AFR population in the Million Veteran Program (MVP) (*n* = 120,839) and found an OR of 1.16 (95% CI = 1.10–1.22, *P* = 1.01 × 10^–8^). We then meta-analyzed the AFR and EUR results (*n* = 577,021) to obtain an OR of 1.18 (95% CI = 1.12–1.24, *P* = 2.37 × 10^–10^). Performing the same variant-phenotype association in the *All of Us* Research Program, we found an AFR-specific (*n* = 36,262) OR of 1.24 (1.09–1.40, *P* = 3.72 × 10^–4^) and an AFR with EUR meta-analyzed (*n* = 139,019) OR of 1.23 (95% CI = 1.09–1.40, *P* = 8.13 × 10^–4^).

To isolate the independent effects of the 3 significant non-*APOL1* G1/G2 variants on renal disease, we assessed conditional associations for the 3 variants in the PMBB AFR population for all phenotypes originally found to be significantly associated during assessment of conditioning for *APOL1* G1/G2, which was risk modeled using APOL’s well-known recessive inheritance pattern (Table 2). The *APOL1* rs2239785 (p.E150K) allele and the *APOL2* rs7285167 (p.R182C) allele were no longer significantly associated with renal phenotypes. However, the *APOL3* stop-gain variant rs11089781 (p.Q58*) remained nominally significantly associated with increased risk for renal disease. Conditioning *APOL3* p.Q58* on *APOL1* p.N264K made minimal difference compared with the unconditional analysis, and conditioning on both *APOL1* G1/G2 and p.N264K resulted in minimal change compared with only conditioning on *APOL1* G1/G2 (Supplemental Table 4). This persistence of the association signal indicates that this stop-gain variant in *APOL3* has some independent effect on CKD risk.

### Interrogation of APOL3 p.Q58* association with ESRD.

To investigate potential gene dosage effects of *APOL3* p.Q58* on ESRD risk, we compared the prevalence of individuals with ESRD among different carrier statuses of rs11089781 in the AFR population in our biobank. 7.3% of noncarriers of *APOL3* rs11089781 were diagnosed with ESRD (cases, *n* = 501; controls, *n* = 6,386) compared with 9.4% of heterozygote carriers (cases, *n* = 332; controls, *n* = 3,206), and 15.5% of homozygote carriers (cases, *n* = 84; controls, *n* = 459). Using Fisher’s exact tests, the prevalence of ESRD was significantly different across all 3 carrier groups (*P* < 1 × 10^–3^), suggesting a gene dosage effect on renal disease. Furthermore, we compared the age of onset for ESRD in our cohort among patients with different carrier statuses of p.Q58*. The average age of onset was 55.0 years in patients who do not carry the variant, 53.1 years in patients who carry 1 copy of the variant, and 49.6 years in patients who carry 2 copies. Using Student’s *t* tests, the difference between noncarriers and monoallelic carriers was near significant (*P* = 0.056), the difference between monoallelic and biallelic carriers was nominally significant (*P* = 0.042), and the difference between noncarriers and biallelic carriers was most significant (*P* = 0.002).

Building upon our PheWAS results, we analyzed relevant EHR-derived laboratory measurements and kidney imaging traits to perform quantitative associations with this *APOL3* stop-gain variant. Focusing on renal and kidney-related hematological lab values, we computed the maximum, median, and minimum values for each trait for each individual, performed inverse-normal transformation, and again ran all associations in the PMBB AFR population. The same analysis was carried out for relevant kidney imaging traits, where we curated clinically available CT scans from patients in PMBB, segmented the left and right kidneys, extracted quantitative imaging traits, and computed the maximum, median, and minimum values for each trait for each individual followed by normalization. We found that the *APOL3* variant was strongly associated with decreased minimum estimated glomerular filtration rate (eGFR; *P* = 2.17 × 10^–7^, *n* = 10,435) and increased maximum creatinine (*P* = 3.85 × 10^–6^, *n* = 10,435), consistent with increased risk for renal disease (Supplemental Table 5). We also identified nominally significant associations with decreased minimum red blood cell counts (*P* = 3.49 × 10^–3^, *n* = 10,020), consistent with decreased erythropoietin production, as well as decreased minimum lymphocyte percentage (*P* = 5.57 × 10^–3^, *n* = 10,118). We replicated quantitative associations for eGFR and creatinine in MVP and identified concordant significant associations for decreased mean eGFR (*P* = 2.42 × 10^–13^, *n* = 110,674) and increased mean creatinine (*P* = 1.63 × 10^–8^, *n* = 116,531). We observed similar associations in *All of Us* for decreased minimum eGFR (*P* = 7.97 × 10^–3^, *n* = 25,572) and increased maximum creatinine (*P* = 8.13 × 10^–3^, *n* = 25,572). Finally, using our analysis of quantitative kidney-derived CT imaging traits, carriers of the *APOL3* p.Q58* were found to have significantly decreased minimum kidney volume (*P* = 2.49 × 10^–3^, *n* = 1,767) as well as decreased minimum kidney surface area (*P* = 5.14 × 10^–3^, *n* = 1,768) (Supplemental Table 5). Similar association results were observed when the AFR population results were meta-analyzed under a fixed-effects model with results from the EUR population (Supplemental Table 6). Thus, in addition to its association with diagnosis codes reflecting CKD, *APOL3* p.Q58* is associated with a number of quantitative traits concordant with CKD across 3 different cohorts enriched in participants genetically similar to AFR reference populations.

We then analyzed the association of *APOL3* p.Q58* with CKD after stratifying by *APOL1* G1/G2 carrier status. Given the possible recessive effects of our *APOL3* variant suggested by our gene dosage results, we performed the stratification analyses under both an additive and recessive model. Interestingly, we found that *APOL3* p.Q58* increases risk for ESRD most significantly under a recessive inheritance pattern in *monoallelic* *APOL1* G1/G2 risk allele carriers (Tables 3 and 4). We found the same result upon stratified analyses of this variant with eGFR and creatinine. This result suggested that this *APOL3* stop-gain variant may have an epistatic interaction with *APOL1* G1/G2 and increases risk of CKD most prominently in monoallelic carriers for either the *APOL1* G1 or G2 allele. Carrier counts for both *APOL1* G1/G2 and *APOL3* p.Q58* are specified in Supplemental Table 7. In these *APOL1* G1/G2 monoallelic individuals, we found that 7.0% of individuals who are low-risk (Q/Q or Q/*) for p.Q58* under its recessive inheritance pattern were diagnosed with ESRD (cases, *n* = 330; controls, *n* = 4,379) compared with 11.6% of individuals who are high-risk (*/*) for p.Q58* (cases, *n* = 29; controls, *n* = 221) with a significant Fischer’s exact test (*P* = 0.011). In addition, the average age of onset for ESRD in the p.Q58* low-risk group was 56.9 years compared with 50.6 years in the p.Q58* high-risk group (*t* test, *P* = 0.036). Furthermore, we performed an interaction analysis between *APOL1* G1/G2, modeled under its well-known recessive inheritance pattern, and *APOL3* p.Q58*, also under a recessive model given the results of the stratified analyses. We found that the interaction between the variants was nominally significant (*P* = 0.02), with a negative association coefficient (β = –0.15), suggesting that although there is evidence of variant interaction, any risk conferred by *APOL3* p.Q58* is likely overwhelmed by biallelic *APOL1* G1/G2 risk.

## Discussion

The growing scale of genetic association studies has powered the discovery of novel disease variants, increased our understanding of disease pathogenesis, and spurred the development of precision medicine therapeutics (13–15). Yet, of all the GWAS currently compiled in the GWAS catalog, approximately 95% of all GWAS participants are genetically similar to EUR reference populations (16). The lack of population diversity not only limits the study and discovery of non-EUR variants to those with high penetrance and large effect sizes (17–20), but also hinders the generalizability of any GWAS discoveries at risk of further compounding existing health disparities (21–23). Even though using increasingly diverse population cohorts will begin to mitigate these concerns, further enriching a genetic association study specifically for variants common in non-EUR populations may also enhance our ability to uncover new genetic variant associations. It is well-known that 2 coding alleles within the *APOL1* gene, G1 and G2, found almost exclusively in individuals genetically similar to West African populations, contribute substantially to risk for CKD. Taking a genome-first approach, we used a medical biobank enriched in participants genetically similar to AFR reference populations with whole-exome genomic data linked to rich phenotypic data to perform PheWAS of protein-coding variants in the 6 *APOL* genes. After correction for multiple testing, we identified several variants in the *APOL* gene family predominantly represented in the AFR population and significantly associated with CKD. Of particular interest, we identified a stop-gain variant, p.Q58*, in the *APOL3* gene, with an AFR MAF of 0.211 and EUR MAF of <0.001, that is significantly associated with CKD risk independent of *APOL1* G1/G2. These results highlight the value of combining targeted genome-first approaches with PheWAS in diverse patient biobanks for better understanding genetic risk for kidney disease.

Our initial analysis identified 3 significant variants other than *APOL1* G1/G2 with significant kidney disease associations. rs2239785 is a missense variant in *APOL1* that has been previously reported to be linked with risk for nondiabetic nephropathy and focal segmental glomerulosclerosis (24, 25). Another missense variant, rs7285167, in *APOL2* has been shown to be weakly associated with all-cause ESRD (26); additionally, it has been identified in a *APOL2* protein-specific quantitative trait loci study as strongly associated with protein levels (27). However, this variant is in moderate linkage with *APOL1* G1/G2 (*r^2^* = 0.264). Furthermore, in our present study, the significant association signals for both variants disappeared when conditioning on *APOL1* G1/G2 recessive risk, decreasing our confidence that they play an independent role in renal disease risk. A third variant that we identified, rs11089781, is a stop-gain variant in *APOL3* at amino acid position 58 of 402 (p.Q58*), thereby truncating most of the peptide and likely inhibiting its wild-type function. This specific nonsense mutation is also documented as likely to trigger nonsense-mediated decay (28). rs11089781 has previously been identified to be weakly associated with nondiabetic nephropathy in small African American and Hispanic American populations, but the finding did not always replicate successfully (29, 30). This *APOL3* p.Q58* variant is in minimal LD with the APOL1 G1/G2 alleles and upon conditioning on *APOL1* G1/G2, remained nominally significantly associated with CKD. The average age of onset for ESRD in individuals who are homozygous for the *APOL3* variant was significantly younger than that of individuals who do not carry the p.Q58* variant. Furthermore, we replicated this significant association of *APOL3* p.Q58* with CKD in both the MVP and the *All of Us* Research Program. Finally, we found that *APOL3* p.Q58* was significantly associated with a number of quantitative traits associated with CKD, including increased creatinine and decreased eGFR, kidney volume, and surface area.

The APOL3 protein is thought to play a role in pathogen immunity, similar to APOL1, but specifically targeting intracellular pathogens by dissolving their anionic membranes (31). Our genetic data indicate that wild-type APOL3 may play a protective role in CKD, given that the *APOL3* p.Q58* risk variant is very likely to be loss of function and confers increased risk for renal disease. While association results solely in individuals with low-risk *APOL1* G1/G2 genotypes showed that our *APOL3* variant was still strongly associated with increased risk for CKD, our stratified analyses identified that *APOL3* p.Q58* contributes the most risk for renal disease in patients who carry *1 copy* of an *APOL1* G1 or G2 risk allele, suggesting a complex epistatic interaction with *APOL1* G1/G2. Of interest, there is an increasing body of evidence suggesting that *APOL1* G1 and G2 are toxic gain-of-function mutations, despite their observed recessive inheritance pattern (9, 32–34). A previous study on the interactions between APOL1 and APOL3 suggested that deletion of *APOL3* triggers intracellular actomyosin reorganization, increasing susceptibility to kidney disease through *APOL1*-induced podocyte dysfunction and kidney damage (35). The specific mechanisms by which *APOL1* risk alleles induce podocyte dysfunction are still being studied, with evidence that suggests the *APOL1* variant proteins increase endoplasmic reticulum stress, enhance inflammatory signaling within the cells, and interfere with endosomal trafficking (36–38). The fact that *APOL3* p.Q58* contributes no additional significant risk in individuals who carry 0 copies of *APOL1* G1/G2 suggests that the truncated APOL3 protein is not likely to have a toxic gain of function and further supports the presence of some epistatic interaction with the *APOL1* variants. The lack of additional significant risk in patients with 2 *APOL1* G1/G2 risk alleles may indicate that, in this high-risk situation, loss of APOL3 has little effect on further increasing CKD risk. Our interaction analysis also showed a nominally significant association between the interaction of *APOL1* G1/G2 and *APOL3* p.Q58* with CKD, further supporting the presence of some variant-variant interaction. The negative association coefficient indicates that the overall effect of the interaction term is less than the cumulative effects of the 2 separate *APOL1* and *APOL3* risk alleles, supporting the conclusion from the stratified analyses that this truncating *APOL3* variant confers little additional risk in the setting of the high-risk *APOL1* G1/G2 genotype. The concept that loss of *APOL3* promotes the toxic gain of function of a *single* copy of *APOL1* G1/G2 might partially explain the observed positive selection of this *APOL3* stop-gain variant in AFR populations, inferred from the regions of extended homozygosity around the truncating mutation (39). A potential explanation is that selection for the *APOL3* loss-of-function variant may increase the pathogenicity of *APOL1* G1/G2, increasing potential for efficacy against *Trypanosoma* infection, even in the presence of only 1 G1 or G2 allele. We suggest that the protective effects of wild-type APOL3 on CKD, most markedly observed in monoallelic *APOL1* G1/G2 carriers, contribute to the general lack of kidney injury induced by 1 copy of the *APOL1* risk alleles, but not by 2 copies of the *APOL1* risk alleles. This gives rise to the biological manifestation of a recessive inheritance pattern for the *APOL1* G1/G2 alleles while reconciling with their gain-of-function toxicity.

The genome-first approach used in our study to filter for AFR-specific protein-altering variants allowed us to identify and focus on variants that likely would have been omitted in larger association studies in EUR-dominated populations. Many of the variants included in our study are extremely rare in the EUR population (Supplemental Table 1), including this *APOL3* stop-gain variant, and are too rare to study in predominantly EUR cohorts, highlighting the necessity of study cohorts enriched for non-EUR populations. Furthermore, the threshold for significance used in our initial meta-analysis was calculated based on a strict Bonferroni’s correction of the total number of genotype-phenotype associations performed, often recommended for PheWAS (40). However, it is evident that not all the variants studied in the *APOL* gene family are in perfect linkage equilibrium and not all phenotypes analyzed are independent of each other. Using an overly strict significance threshold gives us increased confidence in our findings.

We note that there are certain limitations in the context of our work. Phenotyping data derived from EHRs have intrinsic noise and imprecisions. We attempted to mitigate this by using Phecodes that group relevant ICD codes together and applying strict quality control steps on our quantitative clinical traits. We also recognize that there is some selection bias, in that patients with disease are more likely to get laboratory markers measured and to undergo CT imaging. This means that the quantitative traits derived from lab values and imaging metrics we have available may be enriched for individuals with pathological trait values. However, this problem is reduced by the diversity in phenotypes in the PMBB, such that a large proportion of patients have nonrenal conditions and may still have normal kidney-related lab values and imaging traits. In addition, our LD calculations were based on unphased WES data estimated using maximum likelihoods on the haplotype frequency cubic equation instead of phased haplotype frequencies. Although the computed values may be inexact, we also obtained LD metrics calculated using phased haplotypes from the 1000 Genomes Project as validation. While the overall sample size of the AFR population in PMBB is limited, we replicated our observations in MVP and *All of Us*, both of which have substantial participation from individuals genetically similar to AFR reference populations. However, it is worthwhile to note that the reference populations used for defining population assignments differ between the biobanks, as noted in the Methods.

In conclusion, our study represents a targeted approach to studying population-specific genetic variation in a diverse medical biobank. Our approach identified multiple AFR-specific protein-altering variants in the *APOL* gene family implicated in kidney disease risk, including a stop-gain variant in *APOL3* that increases the risk of CKD primarily in persons carrying 1 *APOL1* G1/G2 risk allele. While it is imperative that population diversity is emphasized when recruiting patients for biobanks and building cohorts for association studies, our genome-first approach that filters for population-specific variants represents a step in that same direction in helping us understand disease risk in underrepresented populations.

## Methods

### Sex as a biological variable.

Cisgender women and men were included the study.

### Setting and study participants.

All individuals who were recruited for the PMBB are patients of clinical practice sites of the University of Pennsylvania Health System (12). Replication analyses were conducted using genotype and imputed genetic data from the MVP (41) and whole genome sequencing data from the *All of Us* Research Program (42) as well as both of their respective corresponding phenotyping data derived from the EHR.

### Exome sequencing.

This study included 43,731 individuals in the PMBB with exome sequencing and corresponding EHR-derived traits. Genetic sequencing was performed by the Regeneron Genetics Center using protocols as described previously, and all sequences were mapped to GRCh38 (12). In our population-specific and subsequent meta-analyses, we identified individuals genetically similar to the AFR (*n* = 11,198) and EUR (*n* = 30,324) superpopulations using kernel density estimation as defined by HapMap3 (43). Of note, our WES data was unphased, so LD estimates were calculated by finding the maximum likelihood solution of the haplotype frequency cubic equation as implemented by plink 2.0 (44, 45). As validation, LD metrics were also obtained using phased haplotypes from the 1000 Genomes Project (46).

For replication studies in MVP, we interrogated an additional 121,177 AFR individuals and 449,042 EUR individuals with genotyped and imputed data, obtained as previously described (47, 48). Population assignments were based on genetic similarity using a random forest classifier to respective reference populations from the 1000 Genomes Project. In the *All of Us* Research Program, we used 50,080 AFR individuals and 125,860 EUR individuals with whole-genome sequencing data. Population assignments were determined based on genetic similarity using a random forest classifier to respective reference populations from the Human Genome Diversity Project and 1000 Genomes (https://www.internationalgenome.org/data-portal/data-collection/hgdp). Documentation on data quality and curation are as described previously (49, 50).

### Variant annotations.

Annotations for variants selected for the initial analyses in PMBB were obtained using ANNOVAR (51). Variants of interest were annotated as pLOF, missense, or in-frame insertion/deletion variants according to the NCBI Reference Sequencing database (https://www.ncbi.nlm.nih.gov/refseq/). pLOF variants were defined as frameshift substitution, stop-gain, or splicing variants. MAFs for each variant were calculated in the relevant PMBB population using their respective allele counts. Only variants with a MAF of >0.1% in the PMBB AFR population were considered in our study. MAFs for all studied variants were also compiled from the Genome Aggregation Database gnomAD (https://gnomad.broadinstitute.org/) (v4.0.0) (52).

### Phenotype data collection.

ICD-9 and ICD-10 disease diagnosis codes and laboratory measurements were extracted from patient EHRs for the PMBB. Binary phenotypes for each individual were determined by mapping ICD-9 and ICD-10 codes to Phecodes as previously described (53). A rule of 2 was then applied where participants were determined as having a certain disease phenotype if they had the corresponding Phecode diagnosis on 2 or more dates, while phenotypic controls consisted of individuals who never had the Phecode. Individuals with a Phecode diagnosis on only one date were not considered in statistical analyses.

Quantitative laboratory traits were also extracted from patient EHRs for the PMBB. All units were converted to their respective clinical traditional units. After removing outliers (greater than 4 standard deviations from the mean), we recorded the minimum, median, and maximum measurements for each laboratory measurement and each individual to use for subsequent association analyses. For our imaging-derived phenotypes, the kidney was segmented from abdominal and pelvic CT scans using TotalSegmentator (54). Of the 7,946 unique individuals with scans in which the entirety of the kidney was captured and labeled, subsequent quantitative kidney traits such as volume and surface area were derived using *PyRadiomics* (55). Outliers were then removed in a similar fashion and a minimum, median, and maximum value for each trait and individual was computed and used for downstream analysis.

Phecodes were classified in an identical fashion in the MVP cohort. Quantitative traits were extracted as previously described (48). Binary Phecodes and quantitative traits were computed in the *All of Us* cohort using the same methods as the PMBB cohort.

### PheWAS.

Within the AFR and EUR population groups in PMBB, we performed a PheWAS for each of the 62 variants of interest against 1,222 binary phenotypes with at least 20 cases in both the PMBB AFR and EUR population. We used the generalized linear-mixed model framework to account for participant relatedness and unbalanced case-control ratios with the SAIGE package (56). Directly genotyped variants were used for step 1 of SAIGE. Whole-exome sequenced variants were used for step 2 of SAIGE. Analyses were adjusted for sex, age, age^2^, and 5 population-specific genetic principal components (PCs) in the AFR population and 10 PCs in the EUR population. A fixed-effects meta-analysis was then performed using the inverse variance method for pooling as implemented by the “meta” R package (57). A Bonferroni-adjusted significance threshold was then computed using the total number of associations performed in both genetically inferred population groups. A suggestive significance threshold was also calculated by adjusting 1 by the total number of associations.

Replication for specific variants and phenotypes of interests were also performed using SAIGE in MVP, adjusting for age, sex, and 10 population-specific PCs (48). Identical methods were used for replication in the *All of Us* cohort as in PMBB, including using SAIGE for association analyses.

### Conditional and stratified analysis.

Summary statistics for conditional associations were calculated from the conditional normal distribution implemented by the SAIGE package (56). Recognizing that a single haplotype would never carry both G1 and G2 (6), we collapsed the alleles into a single risk allele based on the total number of G1/G2 alleles an individual carries. We coded all monoallelic G1/G2 allele carriers as homozygous reference allele carriers given the recessive nature of the *APOL1* G1/G2 risk alleles. Identical variables were adjusted for as in the unconditioned associations.

The same single risk allele that represents *APOL1* risk was used to stratify the study population for our stratified analyses. Associations were also performed using SAIGE and adjusted for the same variables. Both an additive and a recessive inheritance pattern were used to model rs11089781 risk. Interaction analyses were performed in R, using the same recessive inheritance models and adjusting for the same covariates.

### Statistics.

Generalized linear-mixed models were used for all association studies, including conditional and stratified, to account for participant relatedness and unbalanced case-control ratios as implemented by the SAIGE package (56). Subsequent meta-analysis was performed under a fixed-effects model using the “meta” R package (57). Statistical tests including the 2-tailed Student’s *t* test, Fischer’s exact test, and the χ^2^ test were performed in R. A strict Bonferroni’s correction was applied to all PheWAS summary statistics to determine the threshold for significance and correct for multiple hypothesis testing. *P* < 6.63 × 10^–7^ was considered significant.

### Study approval.

Appropriate consent was obtained from each participant regarding storage of biological specimens, genetic sequencing, access to all available EHR data, and permission to recontact for future studies. The study was approved by the Institutional Review Board of the University of Pennsylvania and complied with the principles set out in the Declaration of Helsinki.

### Data availability.

All summary statistics for significant variant-phenotype associations in the PMBB, as well as significant replications from each replication cohort, are fully detailed in the main text and in Supplemental Tables 2–6. A list of all the single variants used in this study is provided in Supplemental Table 1. All other relevant data values can be found in the Supporting Data Values file. Individual-level data are not publicly available due to research participant privacy concerns; however, requests from accredited researchers for access to individual-level data relevant to this article can be made by contacting the corresponding author. Details on defining phenotypes in MVP are as previously described (48). This study also used data from the *All of Us* Research Program’s Controlled Tier Dataset v7, available to authorized users on the Researcher Workbench. Code for defining phenotypes used in PMBB and *All of Us* can be found at https://github.com/davidz987/APOL3_CKD/commit/426ceff58f72e4368497a7f52c97aa5047d52969

## Author contributions

All authors reviewed and approved the submitted version of the manuscript. DYZ, MDR, and DJR conceived the idea, designed the project, acquired the data, and interpreted the results. DYZ performed all the analyses in the PMBB and *All of Us* and wrote the manuscript. MGL and SMD performed all analyses in the MVP. JTD and WRW acquired and processed all the CT imaging data. LGL provided critical guidance on interpreting and communicating results for population-specific findings.

## Supplementary Material

Supplemental data

ICMJE disclosure forms

Supplemental tables 1-7

Supporting data values

## Figures and Tables

**Table 1:**
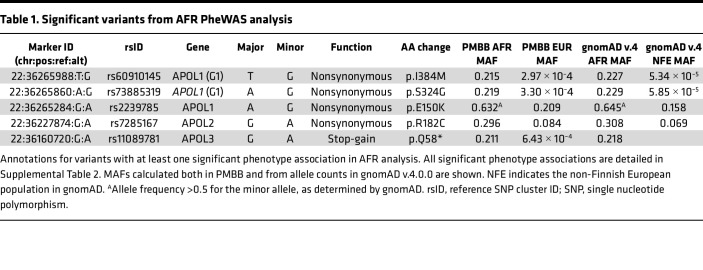
Significant variants from AFR PheWAS analysis

**Table 2:**
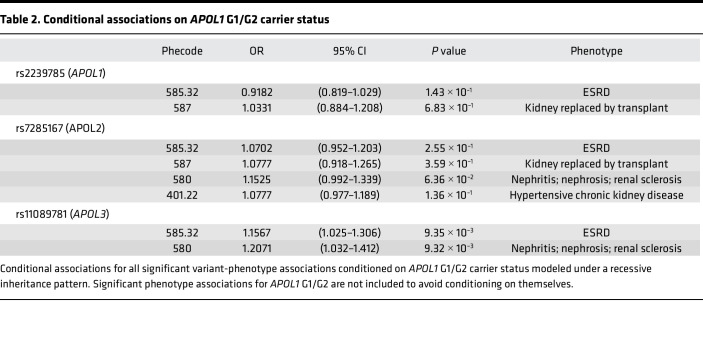
Conditional associations on *APOL1* G1/G2 carrier status

**Table 3:**
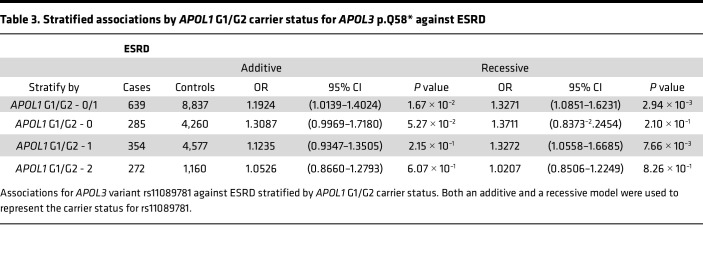
Stratified associations by *APOL1* G1/G2 carrier status for *APOL3* p.Q58* against ESRD

**Table 4:**
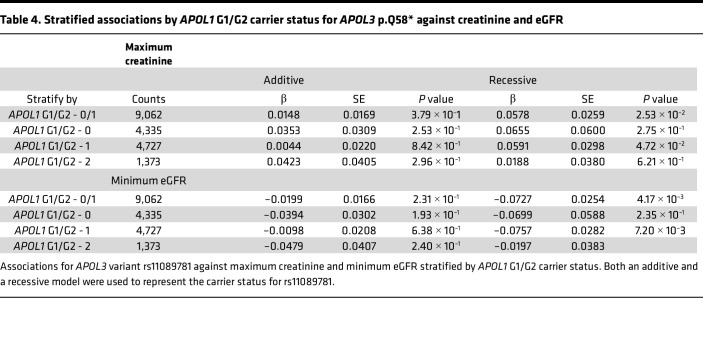
Stratified associations by *APOL1* G1/G2 carrier status for *APOL3* p.Q58* against creatinine and eGFR

## Version 1 08/20/2024

In-Press Preview

## Version 2 10/08/2024

Electronic publication
