# Supplementary material for: Protein-truncating variant in APOL3 increases chronic kidney disease risk in epistasis with APOL1 risk alleles
Source: JCI Insight. 2024 Oct 8;9(19):e181238. doi: 10.1172/jci.insight.181238 (PMC11466179; doi:10.1172/jci.insight.181238)
Supplement: Supplemental data [file jciinsight-9-181238-s143.pdf]

Supplemental Materials

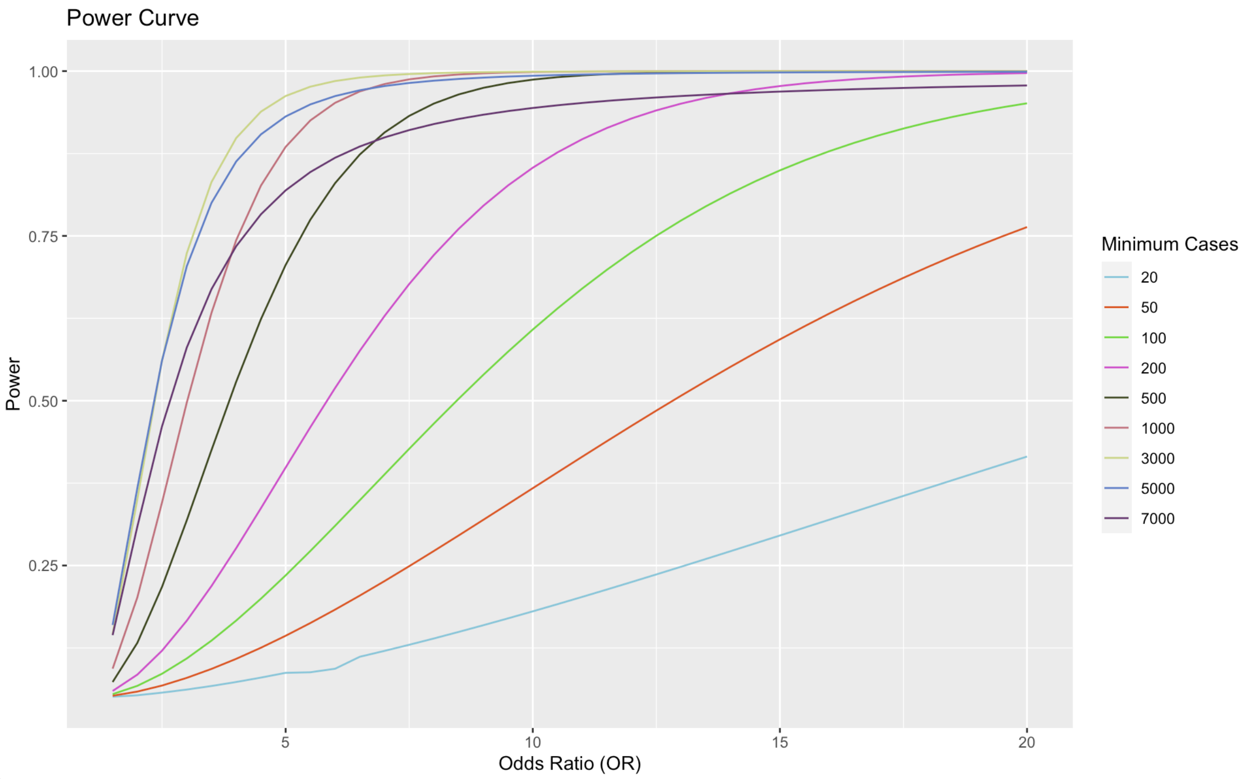

Supplemental Figure 1. Power analysis for detecting significant associations in the PMBB AFR population. We used a MAF threshold of 0.1% in our study cohort (*n* = 11,198). Power curves plotted as a function of effect size with phenotypes of various case counts representative of phenotypes from the PMBB.

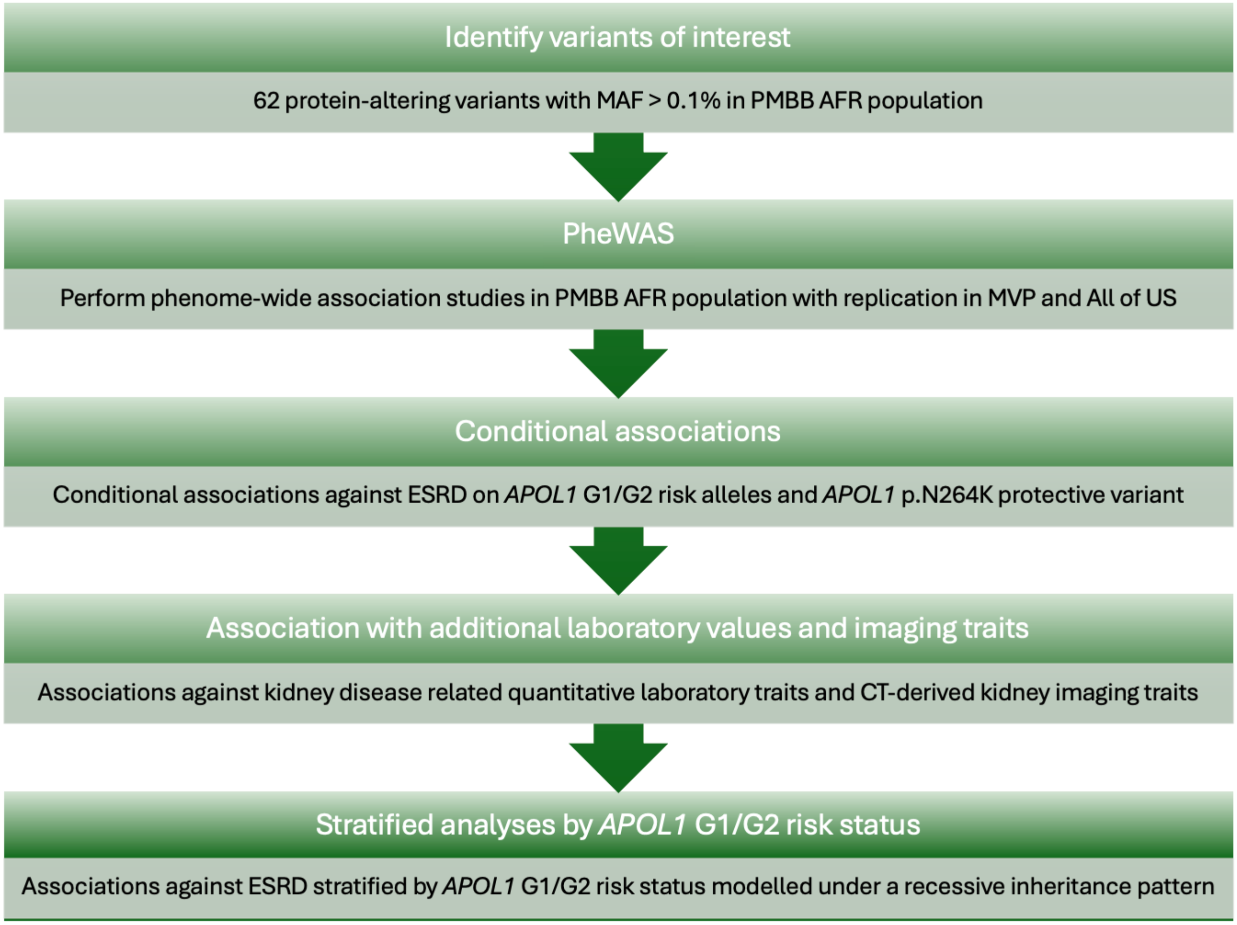

Supplemental Figure 2. Analysis plan. Outline of study design from identifying variants of interest to all downstream association analyses performed.

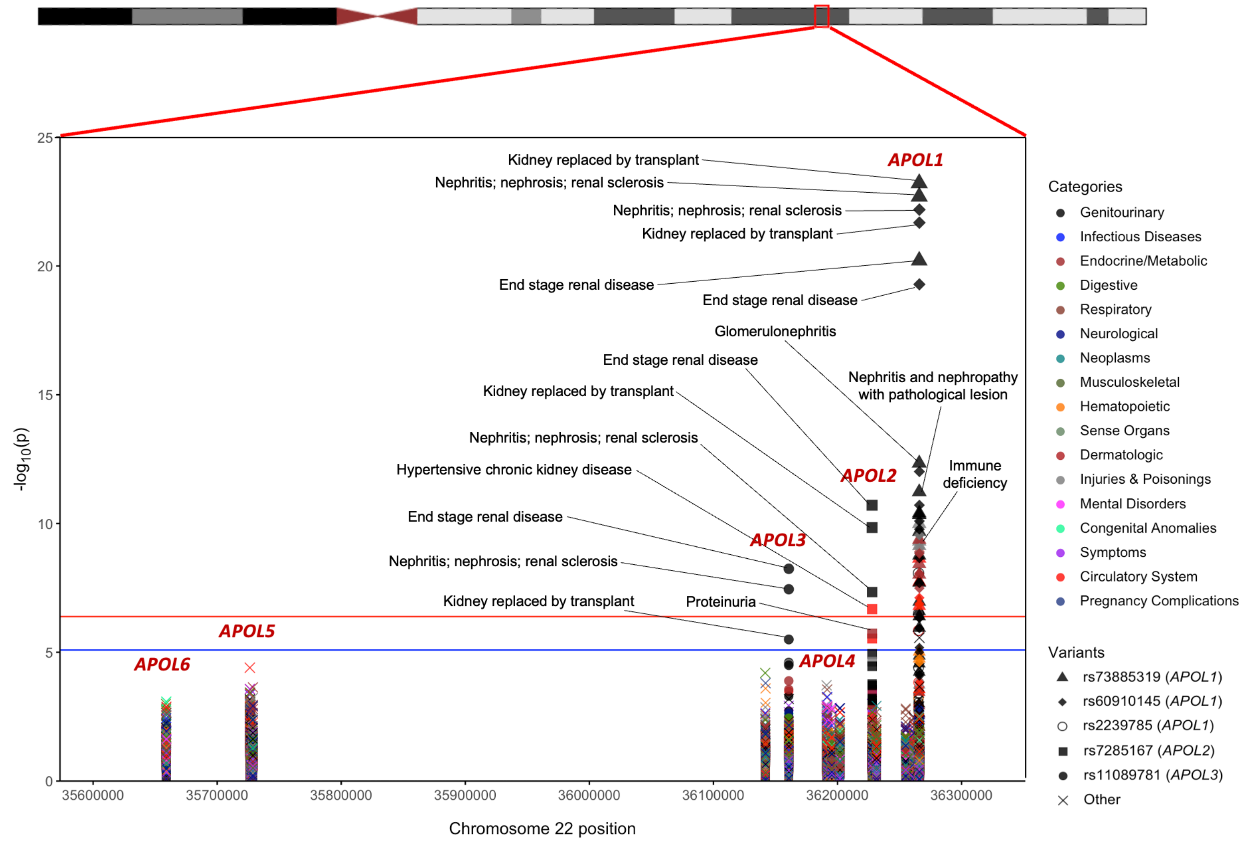

Supplemental Figure 3. Meta-analysis of AFR and EUR PheWAS for 62 protein-altering variants in *APOL* genes. The red line represents the Bonferroni-adjusted *p*-value significance threshold of 4.10E-07. The blue line represents a suggestive *p*-value threshold of 8.21E-06. Variants with at least one phenotype association above the significance threshold are listed in the legend and detailed in Supplemental Table 3.

1KG AFR LD (r^2) (n = 894)						
rs73885319 (*APOL1* G1)	1.000					
rs60910145 (*APOL1* G1)	0.996	1.000				
rs71785313 (*APOL1* G2)	0.052	0.052	1.000			
rs2239785 (*APOL1*)	0.152	0.151	0.053	1.000		
rs7285167 (*APOL2*)	0.215	0.216	0.009	0.089	1.000	
rs11089781 (*APOL3*)	0.035	0.036	0.007	0.031	0.134	1.000
	rs73885319 (*APOL1* G1)	rs60910145 (*APOL1* G1)	rs71785313 (*APOL1* G2)	rs2239785 (*APOL1*)	rs7285167 (*APOL2*)	rs11089781 (*APOL3*)

Supplemental Figure 4. LD heatmap between significant meta-analyzed variants. Includes 5 variants with significant phenotype associations and *APOL1* G1/G2 risk alleles computed using haplotypes from the 1000 Genomes Project using its GRCh38 high-coverage cohort.
